# Supplementary material for: Large off-diagonal magnetoelectricity in a triangular Co2+-based collinear antiferromagnet
Source: Nat Commun. 2023 Dec 5;14:8034. doi: 10.1038/s41467-023-43858-z (PMC10698043; doi:10.1038/s41467-023-43858-z)
Supplement: Supplementary file 1 — Supplementary Information [file 41467_2023_43858_MOESM1_ESM.pdf]

Supplementary Information

Large Off-diagonal Magnetoelectricity in a Triangular Co^{2+}-based Collinear Antiferromagnet

Xianghan Xu[1*], Yiqing Hao[2], Shiyu Peng[3], Qiang Zhang[2], Danrui Ni[1], Chen Yang[1], Xi Dai[3], Huibo Cao[2], and R.J. Cava[1*]

1 Department of Chemistry, Princeton University, NJ 08544

2 Neutron Scattering Division, Oak Ridge National Laboratory, Oak Ridge, TN 37831

3 Department of Physics, Hong Kong University of Science and Technology, Hong Kong, China

*Xianghan Xu: xx8060@princeton.edu

*R.J. Cava: rcava@princeton.edu

Section 1. Derivation of the Curie-Weiss theorem of systems with crystal field interaction

Subsection 1. Atomic model Hamiltonian and eigenstates

The definition of each term in the atomic Hamiltonian in Sec. vi is defined here,

$$\hat{H}_{kanamori} = \frac{U}{2}\sum_{a,\sigma\neq\sigma'}\hat{n}_{a\sigma}\hat{n}_{a\sigma'} + \frac{U'}{2}\sum_{a\neq b,\sigma\neq\sigma'}\hat{n}_{a\sigma}\hat{n}_{b\sigma'} + (U'-J)\sum_{a>b,\sigma}\hat{n}_{a\sigma}\hat{n}_{b\sigma} - J\sum_{a\neq b}\hat{c}^{\dagger}_{a\uparrow}\hat{c}^{\dagger}_{b\downarrow}\hat{c}_{b\uparrow}\hat{c}_{a\downarrow} - J\sum_{a\neq b}\hat{c}^{\dagger}_{a\uparrow}\hat{c}^{\dagger}_{a\downarrow}\hat{c}_{b\uparrow}\hat{c}_{b\downarrow}$$

$$\hat{H}_{cf} = \sum_{ab\sigma\sigma'}\Delta_{a\sigma,b\sigma'}\hat{c}^{\dagger}_{a\sigma}\hat{c}_{b\sigma'}$$

$$\hat{H}_{soc} = \lambda_{soc}\hat{L}\cdot\hat{S}$$

where a (b) and σ (σ') represent orbital and spin index respectively and Δ means CFI elements subject to C_3 point group for paramagnetic state. U and J are strength of Coulomb interaction and Hund's coupling as well as the parameter $U' = U - 2J$.

After diagonalizing the atomic Hamiltonian in Fock subspace of seven electrons as

$$H_{atom}\left|\Gamma_i\right\rangle = E_i\left|\Gamma_i\right\rangle$$
$$\left|\Gamma_i\right\rangle = \sum_{j=1}^{120}\left|I_j\right\rangle U_{I_j,\Gamma_i} \quad i = 1, 2, (, 3, 4, \cdots)$$

where E^i is the atomic eigen-energy and $|\Gamma_i >$ is the atomic eigenstate. Here we denote the ground states as $|\Gamma_1 >$ and $|\Gamma_2 >$.

Subsection 2. Principal axis

The total magnetic moment operator is defined as

$$\hat{M} = \frac{\hat{L} + g\hat{S}}{\hbar}$$

where $g = 2$ for electron's spin and $\hbar$ will be neglected hereafter for simplicity. When projecting the total magnetic moment operator onto the ground state subspace, we obtain its 2×2 matrix representation (M). In the pseudo-spin space, there are naturally three Pauli matrices (σ_i). Then we can expand M using σ_i as,

$$M_m = \lambda_{mj}\sigma_j \quad \forall m \in \{x, y, z\} \quad \text{and} \quad j \in \{x', y', z'\}$$

where x, y, z means the direction in real space and x', y' and z' shows the direction in pseudo-spin space. The λ matrix is actually the g-factor, which is a general 3×3 matrix here rather than the diagonal form. Then SVD can be used for decomposing λ matrix,

$$\lambda = U\Sigma V^T$$

and then we can define some new directions for both M and σ_i, like,

$$\tilde{M} = U^T M$$
$$\tilde{\sigma} = V^T \sigma$$

Therefore,

$$\tilde{M}_m = \Sigma_j \tilde{\lambda}_{mj}\delta_{mj}\tilde{\sigma}_j \quad \forall m \in \{\tilde{x}, \tilde{y}, \tilde{z}\} \quad \forall j \in \{\tilde{x}', \tilde{y}', \tilde{z}'\}.$$

Now we have three new directions, called principal axes here. Actual analysis for any direction can be performed easily.

Subsection 3. Curie-Weiss theorem

Here we take the principal-x axis for example and similar for any other direction. The total Hamiltonian for the pseudo-spin system with uniform external magnetic field reads,

$$H_{total} = H_{atom} + H_{zeeman} = \begin{pmatrix} E_1 & 0 \\ 0 & E_1 \end{pmatrix} - \mu_B \tilde{\lambda}_{\tilde{x}\tilde{x}'} \tilde{\sigma}_{\tilde{x}'} B_{\tilde{x}} \sim -\mu_B \tilde{\lambda}_{\tilde{x}\tilde{x}'} \tilde{\sigma}_{\tilde{x}'} B_{\tilde{x}}$$

By diagonalizing it, two eigenvalues will be obtained: $\pm\mu_B B_{\tilde{x}} m_1$.

Then the partition function is defined as,

$$Z = Tr\left(e^{-\frac{H}{k_B T}}\right) = e^{\frac{\mu_B m_1}{k_B T} B_{\tilde{x}}} + e^{-\frac{\mu_B m_1}{k_B T} B_{\tilde{x}}}$$

the magnetic moment is defined as,

$$M = \frac{k_B T}{Z} \frac{\partial Z}{\partial B_{\tilde{x}}} = \mu_B m_1 \frac{e^{\frac{\mu_B m_1}{k_B T} B_{\tilde{x}}} - e^{-\frac{\mu_B m_1}{k_B T} B_{\tilde{x}}}}{e^{\frac{\mu_B m_1}{k_B T} B_{\tilde{x}}} + e^{-\frac{\mu_B m_1}{k_B T} B_{\tilde{x}}}} = \mu_B m_1 \tanh\left(\frac{\mu_B m_1}{k_B T} B_{\tilde{x}}\right)$$

Note that,

$$\frac{B_{\tilde{x}} m_1 \mu_B}{k_B T} \approx \frac{2meV}{10meV} = 0.25 \text{ at } T = 100K, B_{\tilde{x}} = 10T, m_1 = 3.$$

Hence, it’s good to approximate $\tanh(x) \approx x$, then,

$$M \approx \frac{\mu_B^2 m_1^2}{k_B T} B_{\tilde{x}}$$

Therefore, the magnetic susceptibility is derived as,

$$\chi = \frac{\partial M}{\partial B_{\tilde{x}}}\Big|_{B_{\tilde{x}}=0} = \frac{\mu_B^2 m_1^2}{k_B T} = \frac{C}{T}$$

where $C = \frac{\mu_B^2 m_1^2}{k_B}$.

Finally, using the Weiss proposed molecular field as well as self-consistent mean field method, we can obtain the Curie-Weiss formula with existence of crystal field interaction,

$$\chi = \frac{C}{T - T_c}, \quad C = \frac{\mu_B^2 m_1^2}{k_B}$$

where m_1 is material related and can be obtained from our atomic model simulation. When compared with the molar susceptibility derived from standard Curie-Weiss theorem, it is obvious that the total magnetic moment is:

$$\mu_{eff} = \sqrt{3} m_1$$

After we calculate m_1, the total magnetic magnetic moment is thus straightforward.

Section 2. Supportive data

The Wyckoff positions, atomic coordinates, occupancies, and equivalent isotropic displacement parameters for $CoTe_6O_{13}$ at 300(2) K are provided in Table SI. Relevant crystallographic data are summarized in Table SII. The deposition CSD number is 2242768.

Table SI. **The Crystal structure** Wyckoff positions, atomic coordinates, occupancies, and equivalent isotropic atomic displacement parameters (Å^2) for $CoTe_6O_{13}$. (U_{eq} is defined as one third of the trace of the orthogonalized U_{ij} tensor, space group *R*-3, No. 148, *a* = 10.1660(13) Å, *c* = 18.981(3) Å.

Atoms	Wyck.	*x*/*a*	*y*/*b*	*z*/*c*	Occ.	U_{eq}
Te1	18*f*	0.82234(4)	0.57097(4)	0.73823(2)	1	0.00919(10)
Te2	18*f*	0.60483(4)	0.50405(4)	0.42402(2)	1	0.00817(10)
Co	6*c*	2/3	1/3	0.57732(6)	1	0.0085(2)
O1	6*c*	2/3	1/3	0.7469(3)	1	0.0103(13)
O2	18*f*	0.7561(4)	0.5369(4)	0.64114(18)	1	0.0113(7)
O3	18*f*	0.6144(4)	0.4755(4)	0.52014(19)	1	0.0109(8)
O4	18*f*	0.7951(4)	0.5167(4)	0.4024(2)	1	0.0128(8)
O5	18*f*	0.6670(4)	0.5856(4)	0.7816(2)	1	0.0129(8)

Table SII. Crystallographic data for $CoTe_6O_{13}$ at 300(2) K.

Formula	$CoTe_6O_{13}$
Formula weight	1032.53 g/mol
Crystal system	rhombohedral
Space Group	*R*-3 (No. 148)
Unit cell dimensions	a = 10.1660(13) Å<br>c = 18.981(3)Å
Volume	1698.8(5) $Å^3$
Z	6
Density (calculated)	6.055 g/cm^3
Extinction coefficient	0.00060(3)
Absorption coefficient	16.737 mm^{-1}
F(000)	2658
2θ range	5.100 to 70.200°
Total Reflections	13140
Independent reflections	1660
Refinement method	Full-matrix least-squares on F^2
Final R indices	R_1 = 0.0357; wR_2 = 0.0593
Goodness-of-fit on F^2	1.031

Table SIII. The Rietveld refinement parameters of the lab powder x-ray diffraction on crushed $CoTe_6O_{13}$ crystals at room temperature (290 K). *R*-3 (No. 148), χ^2 = 4.14. The lattice parameters *a* = 10.1593(8) Å and *c* = 18.9739(6) Å.

Atoms	*x*	*y*	*z*	*B*	Occ.	Site
Te1	0.82098(24)	0.57032(33)	0.73853(14)	0.256(36)	1	18*f*
Te2	0.60412(22)	0.50376(23)	0.42402(13)	0.000	1	18*f*
Co1	0.66667	0.33333	0.57727(65)	0.865(249)	1	6*c*
O1	0.66667	0.33333	0.74493(150)	0.000	1	6*c*
O2	0.74975(181)	0.54145(213)	0.64363(87)	0.000	1	18*f*
O3	0.61543(194)	0.47555(207)	0.51820(75)	0.137(476)	1	18*f*
O4	0.80003(224)	0.51626(174)	0.39883(84)	0.000	1	18*f*
O5	0.66347(200)	0.58736(197)	0.78976(99)	1.331(595)	1	18*f*

Table SIV. Wyckoff positions of Co (0, 0, z) and the allowed magnetic moment direction (m_x, m_y, m_z) in *R*-3', *R*-3, *P*-1', and *P*-1 magnetic space groups.

Magnetic space group	Wyckoff positions of Co
***R*-3' (#148.19)**	(0,0,z | 0,0,m_z) (0,0,-z | 0,0,-m_z)<br>(2/3,1/3,z+1/3 | 0,0,m_z) (2/3,1/3,-z+1/3 | 0,0,-m_z)<br>(1/3,2/3,z+2/3 | 0,0,m_z) (1/3,2/3,-z+2/3 | 0,0,-m_z)
***R*-3 (#148.17)**	(0,0,z | 0,0,m_z) (0,0,-z | 0,0,m_z)<br>(2/3,1/3,z+1/3 | 0,0,m_z) (2/3,1/3,-z+1/3 | 0,0,m_z)<br>(1/3,2/3,z+2/3 | 0,0,m_z) (1/3,2/3,-z+2/3 | 0,0,m_z)
***P*-1' (#2.6)**	(0,0,z | m_x,m_y,m_z) (0,0,-z | -m_x,-m_y,-m_z)<br>(2/3,1/3,z+1/3 | m_x,m_y,m_z) (2/3,1/3,-z+1/3 | -m_x,-m_y,-m_z)<br>(1/3,2/3,z+2/3 | m_x,m_y,m_z) (1/3,2/3,-z+2/3 | -m_x,-m_y,-m_z)
***P*-1 (#2.4)**	(0,0,z | m_x,m_y,m_z) (0,0,-z | m_x,m_y,m_z)<br>(2/3,1/3,z+1/3 | m_x,m_y,m_z) (2/3,1/3,-z+1/3 | m_x,m_y,m_z)<br>(1/3,2/3,z+2/3 | m_x,m_y,m_z) (1/3,2/3,-z+2/3 | m_x,m_y,m_z)

Table SV. Intensities of the Bragg peaks measured at 5 K having non-negligible magnetic contributions in single-crystal neutron diffraction. The magnetic contributions F^2_{mag} and nuclear contributions F^2_{nuc} are listed separately.

(*H K L*)	F^2_{obs} at 5 K	F^2_{mag}	F^2_{nuc}	$F^2_{mag+nuc}$
(0 1 -1)	6.63±0.42	7.06	0	7.06
(1 1 -3)	4.21±0.12	4.25	0.44	4.69
(2 1 1)	6.15±0.13	6.04	0.04	6.08
(-1 3 -1)	6.72±0.33	6.04	0.04	6.08
(1 3 1)	4.11±0.23	4.11	0.51	4.62
(2 0 5)	2.80±0.08	2.29	0.13	2.44
(0 -2 5)	2.59±0.07	2.29	0.13	2.44
(0 1 5)	1.02±0.04	0.99	0.01	1.00
(-3 1 -1)	28.77±0.58	5.78	25.71	31.49

Table SVI. NPD peaks having non-negligible magnetic contribution. All of them fall into the reflection condition for magnetic space group *R*-3' (#148.19), which is $2h + k + l = 3n$, and no violated diffractions are observed.

**Index (*h k l*)**	***d* (Å)**
(1 0 1)	7.9677
(0 2 1)	4.2762
(1 1 3)	3.9541
(2 1 1)	3.2681
(0 3 3)	2.6552
(1 2 5)	2.4970
(1 3 1)	2.4147

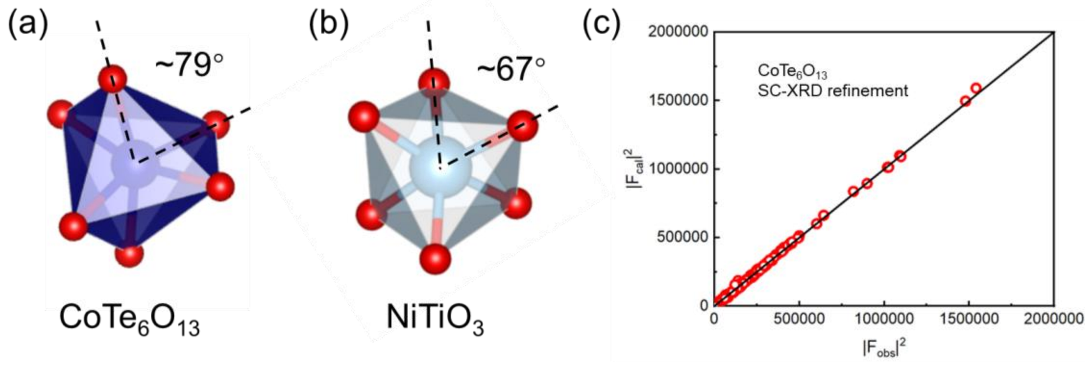

Figure S1. The distorted transition metal octahedra (a - b) The comparison of the octahedron distortion magnitudes of [CoO_6] in $CoTe_6O_{13}$ (a) and [NiO_6] in $NiTiO_3$. The plane of the figure is perpendicular to the local 3-fold axis. Note that the marked angle is supposed to be 60° for an undistorted octahedron. (c) In the SC-XRD refinement result, the calculated intensity (F_cal) closely matches the observed intensity (F_obs), affirming the validity of the adopted lattice structure for $CoTe_6O_{13}$.

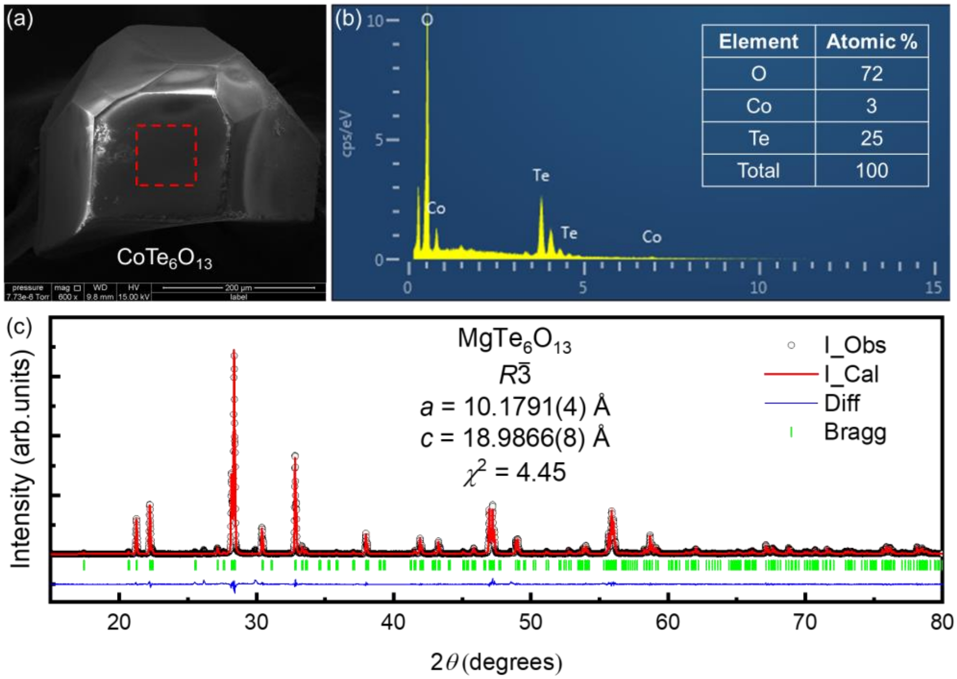

Figure S2. **SEM of $CoTe_6O_{13}$ and XRD of the non-magnetic reference $MgTe_6O_{13}$** (a) The SEM image of a $CoTe_6O_{13}$ single crystal. (b) The EDS spectrum collected in the area marked by the red rectangle in (a). The inset table displays the atomic percentage of elements. The obtained ratio of Co : Te = 0.12 is within error of the Co : Te = 0.1667 ratio in the nominal composition $CoTe_6O_{13}$. The deviation in oxygen content from the nominal ratio is mainly due to the fact that the atomic number of oxygen is small, and the crystal is very insulating. (c) The lab XRD pattern and Rietveld refinement of the non-magnetic isostructural $MgTe_6O_{13}$ polycrystalline sample.

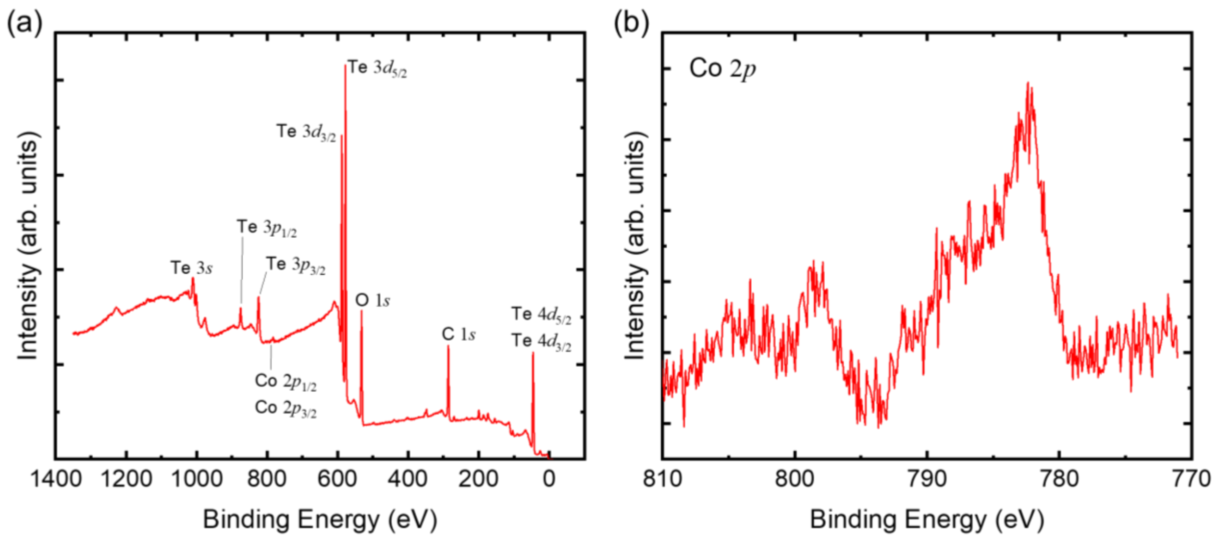

Fig S3. The X-ray photoelectron spectroscopy (XPS) analysis of a $CoTe_6O_{13}$ crystal (a) The entire spectrum. (b) The selected binding energy range for Co 2*p* electrons. Comparison of (b) with the standard reference confirms the 2+ oxidation state of cobalt in $CoTe_6O_{13}$.

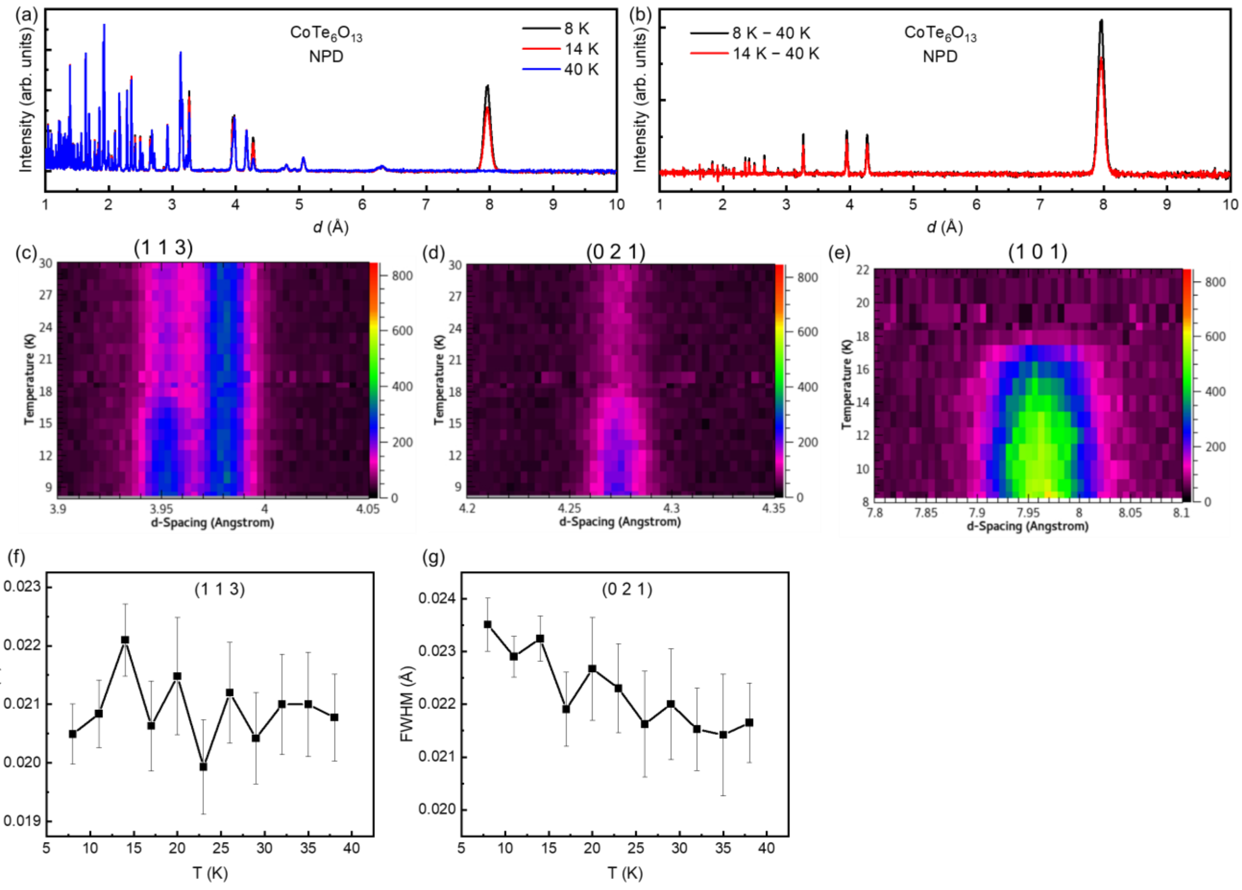

Figure S4. Temperature evolution of the neutron powder diffraction (a) Neutron powder diffraction patterns taken on $CoTe_6O_{13}$ at 8 K, 14 K, and 40 K. (b) Magnetic peaks at 8 K and 40 K obtained by subtracting the 40 K pattern from the 8 K and 14 K patterns, respectively. (c)-(e) Temperature contour plots from a ramping experiment of (1 1 3), (0 2 1), and (1 0 1) peaks, respectively. (f)-(g) FWHM of (113) and (021) peaks as a function of temperature. The slicing interval of temperature is 3 K. Error bars represent standard deviation of the FWHM from Gaussian fitting.

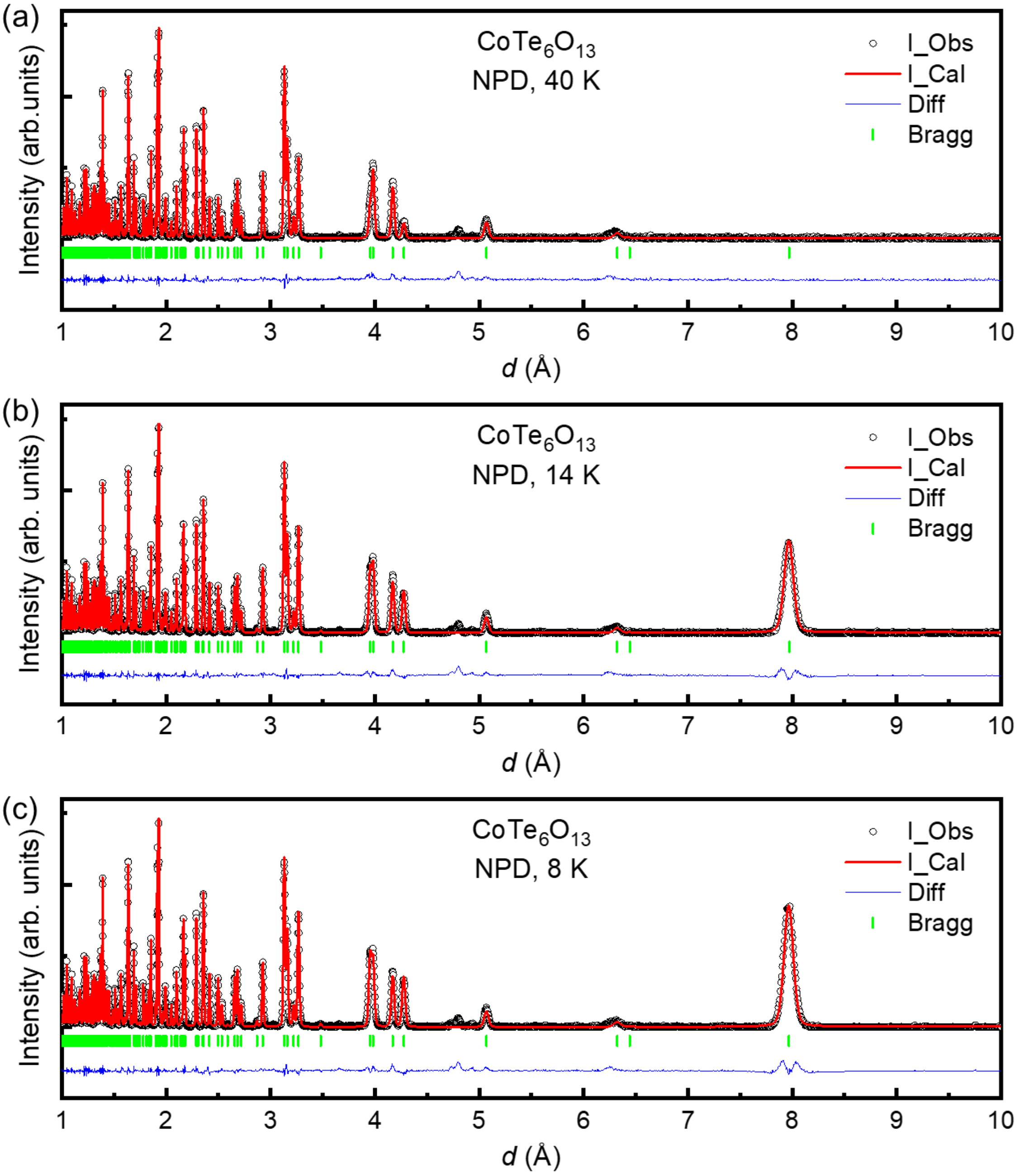

Figure S5. Neutron powder diffraction refinements (a - c) Refinement results of neutron powder diffraction patterns at (a) 40 K, (b) 14 K, and (c) 8 K using the *R*-3′ model. The obtained ordered magnetic moment (m_z) of Co^{2+} is 3.81 μ_B at 14 K and 4.43 μ_B at 8 K.

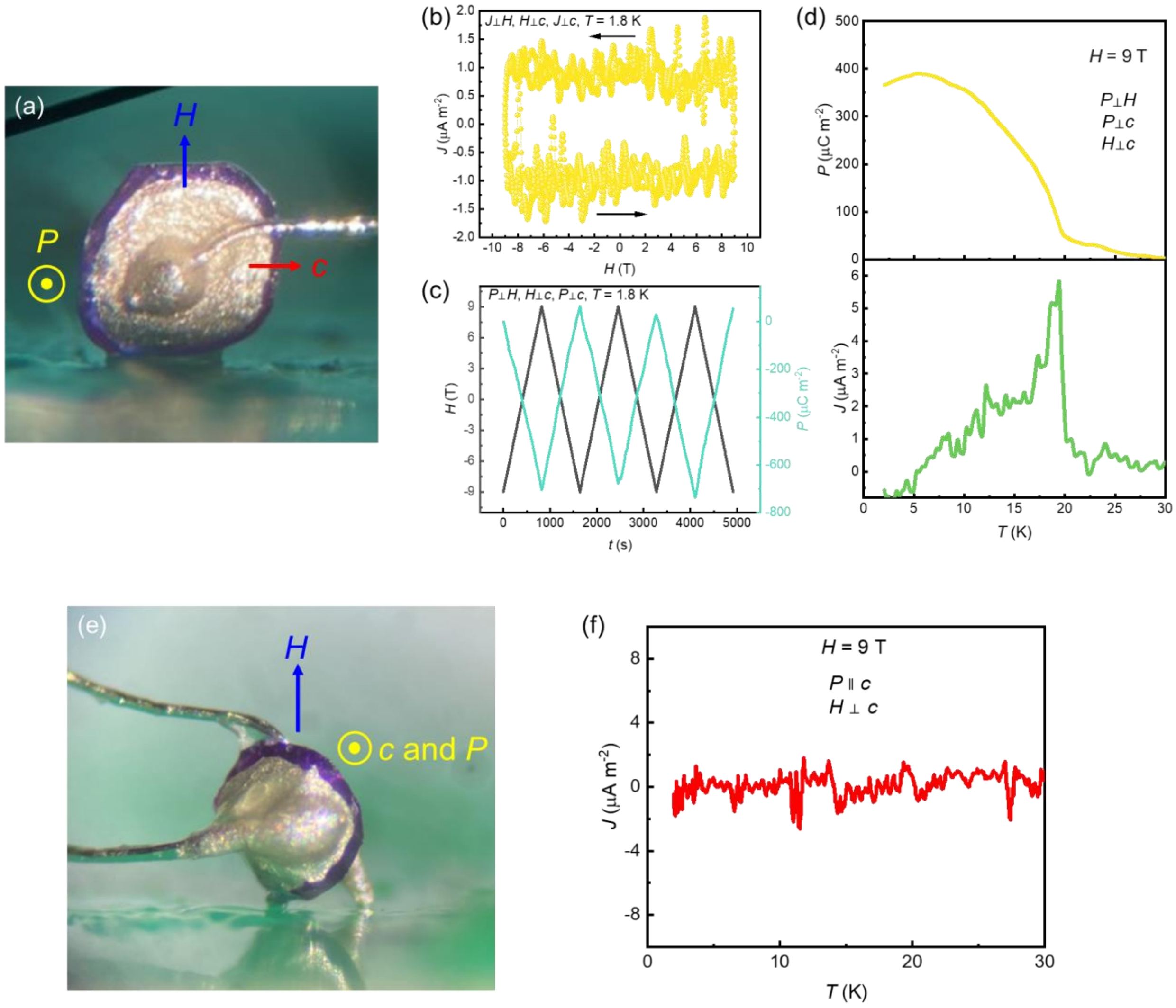

Figure S6. **The magnetoelectric tensor elements σ_{12}** (a - d) **and σ_{13}** (e - f) **measurements.** (a) A photo of the single crystal plate with electrodes used for σ_{12} measurement. Directions of measured polarization, applied magnetic field, and *c* axis are marked by yellow, blue, and red arrows, respectively. (b) Magnetoelectric current at 1.8 K measured with magnetic field perpendicular to the current, and both magnetic field and current are perpendicular to the *c* axis. (c) The sweeping magnetic fields and electric polarizations obtained from (b) as a function of time. (d) The pyroelectric current (lower panel) and obtained polarization (upper panel) as a function of temperature in σ_{12} setup. (e) A photo of the single crystal plate with electrodes used for σ_{13} measurement. (f) The pyroelectric current as a function of temperature in the setup for σ_{13} measurement, which does not show detectable anomaly. Note that the current signals measured on the single crystals are noisier than those on the polycrystalline sample (Fig. 7 in the main text) because the area of the single crystal sample is limited by its smaller size.

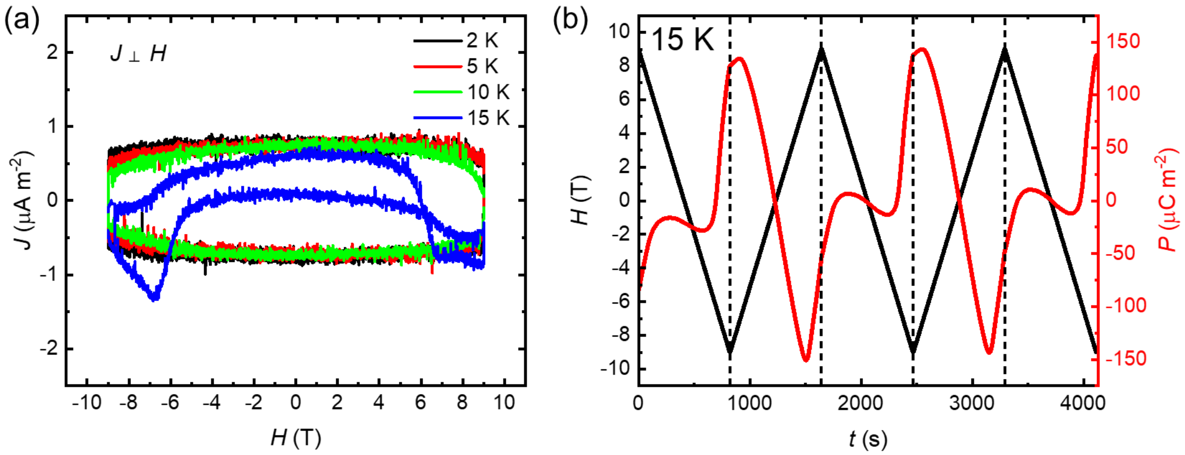

Figure S7. Magnetoelectric response at different temperatures (a) Off-diagonal magnetoelectric current measured at different temperatures (2 K, 5 K, 10 K, and 15 K) on a $CoTe_6O_{13}$ polycrystalline pellet. (b) The polarization as a function of time at 15 K.
